# Method for simultaneous determination of three cooling agents in aerosols by GC–MS

**DOI:** 10.3389/fchem.2025.1699107

**Published:** 2025-10-31

**Authors:** Tinghao Chen, Chenfeng Hua, Chengjie Ma, Bin Peng, Pingping Shang, Ge Zhao, Quanping Yan, Fuwei Xie

**Affiliations:** Key Laboratory of Tobacco Chemistry, Zhengzhou Tobacco Research Institute of China National Tobacco Corporation (CNTC), Zhengzhou, China

**Keywords:** menthol, cooling agent, aerosol, gas chromatography-mass spectrometry, method validation

## Abstract

A cooling sensation is primarily elicited by cooling agents through activation of cold-sensitive receptors such as TRPM8 and TRPA1. Coolants are widely used as functional additives in various industries including food, personal care, pharmaceuticals, and tobacco. In this study, a gas chromatography–mass spectrometry (GC-MS) method was developed to quantify three representative cooling agents—menthol, WS-3(N-ethyl-2-(isopropyl)-5-methylcyclohexanecarboxamide), and WS-23 (2-Isopropyl-N,2,3-trimethylbutyramide)—in aerosol samples. The test aerosols were generated from laboratory-formulated e-liquids under optimized conditions. Aerosols were obtained from an electronic vaping device manufactured by RELX (China). The results demonstrated that: (1) the analytical method exhibited good linearity (*R* ^2^ ≥ 0.9994), with limits of detection (LOD, from 0.137 ng/mL to 0.114 μg/mL), limits of quantification (LOQ, from 0.456 ng/mL to 0.380 μg/mL), relative standard deviations (RSDs, 1.40%–4.15%), and spiked recovery rates (from 91.32% to 113.25%) all meeting the requirements of analytical validation; (2) the cooling agents were detected in both gas and particle phases of the aerosol, with the concentrations in gas-phase being significantly lower than those in the particle phase due to aerosols condensation. Specifically, the gas-phase proportions of menthol, WS-23 and WS-3 ranged from 1.94% to 5.72%, 0.03%–0.08%, and 0.10%–0.18%, respectively. Therefore, the developed GC-MS method satisfies methodological validation criteria and is suitable for application to commercial aerosol samples. It provides a reliable analytical foundation for studying sensory perception of cooling agents under aerosol exposure and offers more precise guidance for their use.

## 1 Introduction

Cooling agents are important additives used to modulate the sensory perception of a wide range of consumer products. Over the past several decades, numerous cooling agents have been developed and extensively applied in the food, pharmaceutical, personal care, cosmetic, and tobacco products (Shen et al., 2009). Among them, menthol, WS-23 (2-Isopropyl-N,2,3-trimethylbutyramide), and WS-3(N-ethyl-2-(isopropyl)-5-methylcyclohexanecarboxamide) are the three most commonly used representatives. In the food sector, the minty cooling sensation is widely utilized in chewing gums, candies, and beverages. This cooling effect invigoration and refreshment, significantly enhancing the consumer experience. In the personal care industry, cooling agents are incorporated into various products such as toothpastes, mouthwashes, shampoos, and mosquito repellents. Notably, combining menthyl lactate with TK -10 markedly improves the perception of skin cooling in cosmetic formulations (Erman, 2004). Furthermore, adding cooling agents in cosmetics also facilitates the dissolution of highly hydrophobic fragrance compounds in aqueous solutions (Larcinese-Hafner and Tchakalova, 2018). In the pharmaceutical field, cooling agents are also incorporated into formulations for anti-pruritic and antiseptic purposes. They mitigate drug-induced irritation and serve as local anesthetics to relieve pain (Liu et al., 2005; Patel et al., 2002). Moreover, combining WS-3 with other synthetic coolants (e.g., Frescolat MGA, TK-10)enhances user experience in cough relief products (Erman, 2004). In the tobacco industry, cooling agents are employed to reduce the harshness of smoke and smooth the inhalation experience. They mask the physiological irritation caused by smoke, which helps alleviate smokers’ psychological discomfort and improve the attractiveness of electronic cigarettes (Anderson, 2011; Tackett et al., 2025). Cigarettes containing cooling agents have thus gained considerable popularity in global markets. For example, mentholated capsule cigarettes achieved an approximate 15% increase in market share in countries such as South Korea, Chile, Peru, and Guatemala. In the U.S., the market share of mint-flavored e-cigarettes doubled between 2017 and 2021 (Ali et al., 2024; Emond et al., 2018; Kim et al., 2018). Moreover, several studies have shown that cooling agents can increase respiratory depth via respiratory inhibition. They also stimulate cold receptors in the nasal cavity to increase alertness. In addition, activation of oropharyngeal cold receptors helps reduce sensations of thirst (Eccles, 2000; Orani et al., 1991; Plevkova et al., 2013; Sekizawa et al., 1996).

Currently, the application of cooling agents in aerosolized form is predominantly associated with tobacco products. In the past 10 years, a variety of analytical techniques have been employed to detect these compounds, including headspace gas chromatography (HS-GC), gas chromatography with flame ionization detection (GC-FID), high-performance liquid chromatography (HPLC), comprehensive two-dimensional gas chromatography coupled with time-of-flight mass spectrometry (GC × GC-TOFMS), and gas chromatography–mass spectrometry (GC-MS) etc. Among these, GC-MS has gained widespread use due to its excellent selectivity, high sensitivity, and short analysis time (Sun et al., 2017; Wang et al., 2020). In 2013, Meruva et al. (2013) employed GC-MS to quantitatively determine the menthol content in mainstream smoke from individual cigarettes. In 2016, Krüsemann and colleagues (Krüsemann et al., 2016) applied headspace GC-MS to detect menthol, menthone, and menthyl acetate in cigarette smoke, and further quantified menthol odor recognition by correlating chemical concentrations with sensory threshold values. In 2017, Sun Haifeng et al. (Sun et al., 2017) developed a method using absolute ethanol extraction to analyze cooling agents in both tobacco smoke and tobacco material. More recently, in 2022, Jabba et al. (2022) perfomed qualitative and quantitative analyses of aerosols from U.S. flavored e-cigarette products using GC-MS and GC-FID. They found that most flavored e-cigarettes added menthol, WS-23, or WS-3 as cooling agents. They also showed that the transfer efficiency from e-liquid to the aerosol was nearly 100%. Furthermore, the study used a Margin of Exposure (MOE) model to assess the inhalation safety of WS cooling agents. The results indicated that under low, medium, and high usage scenarios, exposure levels in most products approached or exceeded regulatory concern thresholds. Recently, headspace sampling and solid-phase microextraction (SPME) are most widely used technique in the literature for the aerosol analysis of e-cigarettes, meanwhile liquid chromatography has gained significant relevance in e-liquid studies (Toledo et al., 2025).

While the increasingly popular cool flavored e-cigarettes among young people, the inhalation toxicology and exposure risks of aerosol coolants are still unclear and worth establishing relevant detection method to promote research (Leventhal et al., 2023). Therefore, besides quantifying cooling agents overall in cigarette smoke, it’s also analytically valuable to study how these compounds are distributed among smoke phases. As early as 1986, W. Holländer and W. Stöber characterized cigarette smoke as a ‘multiphase steady state’ comprising vaporized smoke and a particulate phase. They proposed that the vapor phase and particulate phase exhibit distinct deposition patterns and behaviors within the respiratory tract (Holländer and Stöber, 1986). In 1990, G. Scherer and colleagues demonstrated compositional differences between the particulate and vapor phases of inhaled cigarette smoke (Scherer et al., 1990). Studies in the early 21st century showed that phase composition of smoke aerosols can influence deposition patterns in the respiratory system. These differences affect puffing perception and also the precision in inhalation exposure assessments (Su and Wu, 2004). For example, a higher proportion of free-base nicotine has been shown to increase the perceived smoking harshness or “throat hit” (Chen et al., 2000). Similarly, J. F. Pankow described the gas/particle partitioning coefficient *Kp*as the equilibrium ratio of a compound between the gas and particle phase, which is governed primarily by its volatility. *Kp* thus links vapor pressure to the compound’s tendency to remain in the gas phase versus condense onto particles, and has been associated with throat irritation from smoke inhalation (Pankow, 2001). The research conducted by Plevkova et al. indicates that the vapor form of menthol seems to be more effective in relieving coughing. (Plevkova et al., 2013). In 2015, Tatjana I. Kichko et al. conducted animal experiments and found that the sensory effects of vaporized nicotine are mediated by the complete activation of TRPA1 receptors. This pure effect was shown to be as strong, if not stronger, than the multiple stimuli effects of TPM(Kichko et al., 2015). In 2022, Zeng Shitong et al. demonstrated a correlation among volatility, deposition amount, and sensory irritation of carbonyl compounds in cigarette smoke under oral exposure (Zeng et al., 2022). Wu et al. have shown that gas/particle partitioning significantly affects exposure profiles for nicotine (Wu et al., 2022). Recently, Chandra et al. found that menthol flavored e-liquid produce significantly more micro- and sub-micron particles compared to non-menthol counterparts and is associated with worse lung function indices in cigarette smokers (Chandra et al., 2023). Additionally, regulatory opinions also point out the lack of data for inhalation exposure from specific forms of coolant. However, none of these studies have yet combined quantitative phase-specific measurement of cooling agents with sensory outcomes. This gap motivates the present study (BfR, 2025).

Therefore, current methods that quantify only the total content of cooling agents in aerosolized samples are insufficient. They can not evaluate the cooling sensation more accurately. More comprehensive analytical methods capable of detecting both particle phase and gas phase compositions of cooling agents in aerosols are necessary. This approach bridges the gap in current aerosol-based detection techniques by enabling differentiation of coolants in gas/particle phase. It is not only crucial for cooling sensation evaluation and the application of cooling agents, but also fundamental for risk assessment and product regulations. Different phase distributions lead to different deposition patterns *in vivo*, which in turn can result in varying exposure risks. Consequently, we have developed a method to determine the concentrations of menthol, WS-3, and WS-23 in aerosols. This method simultaneously measures both gas and particle phase content. Therefore, it is suitable for analyzing cooling agent components in electronic aerosol-generating products.

## 2 Materials and methods

### 2.1 Sample collection

**FIGURE 1:**
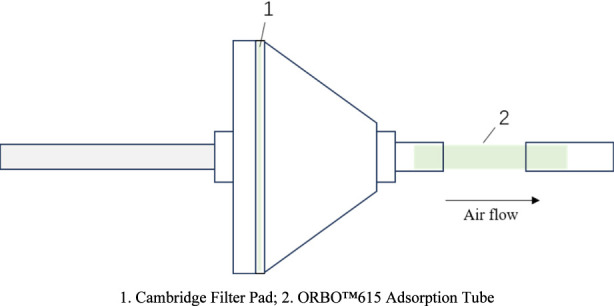
Aerosol collection device.

The electronic cigarette devices were prepared in the laboratory, and e-liquids containing three cooling agents were formulated. Aerosols were captured using a linear smoking machine (Model SM450, Cerulean Ltd., United Kingdom) according to the ISO standard smoking protocol for electronic cigarettes. The puffing mode was set to square-wave with a puff volume of 55 mL, a puff duration of 3 s, and a 30-s interval between puffs. Aerosols from 40 puffs of the e-cigarette device were collected, with the collection process separated into the gas phase and particle phase. The setup is shown in Figure 1.

After collection, the 44 mm Cambridge filter (Körber GmbH, Germany) was removed and placed into a 40 mL amber storage vial, followed by the addition of 30 mL methanol and 750 μL internal standard solution (concentration: 4 mg/mL). The adsorbent from the ORBO™615 tube (Supelco, United States) was transferred to a 12 mL amber storage vial, and 5 mL methanol, 125 μL 1,3-butanediol internal standard solution (4 mg/mL), and 62.5 μL phenylethanone-d8 internal standard solution (40 μg/mL) were added. The mixture was subjected to ultrasonic extraction at 40W for 30 min. The extract from both the gas and particle phases was filtered through a 0.45 μm organic phase membrane (nylon fiber, ANPEL Laboratory Technologies Inc., Shanghai, China) and 1 mL of the filtrate was transferred to a chromatography vial for analysis.

### 2.2 GC-MS analysis

The aerosol cooling agents captured were detected and quantified using a gas chromatography-mass spectrometry system (Model Agilent 7890B/5977A, Agilent Technologies, United States). The chromatographic column used was DB-ALC1 (30 m × 320 μm × 1.8 μm). The injection port temperature was set at 250 °C. The temperature program started at an initial temperature of 100 °C, held for 5 min, then increased at 10 °C/min to 130 °C, held for 5 min, followed by a 5 °C/min increase to 200 °C, held for 0 min, and finally increased at 3 °C/min to 220 °C, held for 3 min. A 1 μL sample was injected with a split ratio of 10:1. The carrier gas was helium (purity ≥99.999%) at a constant flow mode, with a flow rate of 1.5 mL/min.

Mass spectrometry conditions: Solvent delay of 4.3 min, electron ionization (EI) with an ionization energy of 70 eV. The transfer line temperature was 250 °C, and the ion source temperature was 230 °C. The scan mode was set to Selected Ion Monitoring (SIM), and the retention time, quantitative ions, and qualitative ions for the target compounds and internal standards are shown in the Table 1.

**TABLE 1:** Retention times, quantitative ions, and qualitative ions of target compounds and internal standards.

Compounds	Retention Time/min	Quantifier (m/z)	Qualifier (m/z)
Menthol	13.27	138	95,123
WS-23	19.28	114	128,129
WS-3	28.24	100	168
1,3-Butanediol (Internal standard)	4.67	43	72,75
Acetophenone-d8 (Internal standard)	10.42	110	82,128

### 2.3 Thermal Gravimetric analyzer instrument

To optimize the adsorption tube model and validate the reliability of the adsorption tubes used for gas phase collection, a thermogravimetric analyzer (Model TGA 550, Waters Technologies Co., Ltd., Shanghai) was employed to select the optimal adsorption tube from three models: ORBO™615, ORBO™32, and ORBO™90. Different temperature programs were set based on the boiling points and vapor pressures of each cooling agent (Table 2), and mass loss was recorded following the execution of the program.

**TABLE 2:** Temperature program of the thermal gravimetric analyzer.

Compounds	Temperature program	Air flow rate/(mL·min-1)
Menthol	40 °C/min to 50 °C, maintain 10min	500
WS-23	40 °C/min to 70 °C, maintain 10min	500
WS-3	40 °C/min to 80 °C, maintain 10min	500

The adsorbent material from the collected tubes was then transferred into 40 mL amber storage vials, followed by the addition of 20 mL methanol, 500 μL 1,3-butanediol internal standard solution (4000 ppm), and 250 μL phenylethanone-d8 internal standard solution (40 ppm). The mixture was subjected to ultrasonic extraction for 30 min, and the extract was filtered through a 0.45 μm organic phase membrane. A 1 mL aliquot of the filtrate was transferred to a chromatography vial for analysis.

### 2.4 Reagents

All reagents used in this experiment were of analytical grade. Methanol, ethanol, and isopropanol were purchased from Supelco (United States). The three cooling agents were L-menthol (99%, Merck, Germany), WS-23 (>98%, Aladdin Biochemical Technology Co., Ltd., Shanghai), and WS-3 (99%, Merck, Germany). The internal standards used were 1,3-butanediol (99%, McLin Biochemical Technology Co., Ltd., Shanghai) and phenylethanone-d8 (98% atom %D, Beijing Bailingwei Technology Co., Ltd.).

### 2.5 Preparation of standard solutions

Internal standard solutions: 200 mg of 1,3-butanediol and phenylethanone-d8 were each placed in a 50 mL volumetric flask and dissolved in methanol to prepare a 4 mg/mL internal standard solution for particle phase samples. For gas phase samples, 2 mg of 1,3-butanediol and 2 mg of phenylethanone-d8 were dissolved in a 50 mL volumetric flask with methanol to yield a 4 mg/mL internal standard solution.

Mixed standard stock solution: 1 g of menthol, WS-3, and WS-23 were each weighed and placed in a 10 mL volumetric flask and diluted to volume with methanol to prepare individual stock solutions. Then, 1 mL of each individual standard solution was accurately transferred into a 100 mL volumetric flask and diluted with methanol to prepare a 1,000 ppm mixed standard stock solution. For further dilution, 200 mg of menthol, WS-3, and WS-23 were each weighed and placed in a 100 mL volumetric flask and diluted with methanol to prepare individual stock solutions. 100 μL of each individual stock solution was then transferred to a 100 mL volumetric flask and diluted with methanol to obtain a 2 ppm mixed standard stock solution.

Series of working standard solutions: 10, 20, 50, 100, 200, 500, 1,000, 2000, and 5,000 μL aliquots of the 1,000 ppm mixed standard stock solution were transferred to 10 mL volumetric flasks. To each flask, 250 μL of the 4 mg/mL internal standard solution was added, and the volume was adjusted with methanol to prepare particle phase working standard solutions. For gas phase working solutions, 50, 100, 250, 500, 1,000, 2,500, and 5,000 μL aliquots of the 2 ppm mixed standard stock solution were transferred to 10 mL volumetric flasks, and 125 μL of the 40 mg/mL internal standard solution was added before dilution with methanol. The solutions were mixed thoroughly to obtain the working standard solutions for gas phase samples.

### 2.6 Pretreatment conditions optimization

To minimize sample loss, reduce processing time, and simplify the operation steps, the conditions involved in sample preparation were optimized. Among the three solvents—methanol, ethanol, and isopropanol—the solvent with the best extraction efficiency was selected. The effects of solvent extraction volumes of 20–40 mL and 3–5 mL were evaluated for both gas and particle phases. Additionally, the ultrasound extraction power and duration were compared and optimized.

### 2.7 Instrumental analysis methods optimization

Optimization was also carried out for the split ratio and temperature program. To achieve optimal peak shapes, the influence of different split ratios (splitless, 10:1, 20:1, 30:1) on peak shapes, including tailing or flat-top peaks, was assessed. Furthermore, to shorten the analysis time while ensuring good separation of all components, the heating rate and retention times of the temperature program were optimized and adjusted accordingly.

### 2.8 Validation

The methodology was validated by assessing linearity, limits of detection (LOD), limits of quantification (LOQ), precision, and recovery. A total of 6-7 concentration points were selected, and calibration curves for both the gas phase and particle phase were constructed by plotting the ratio of the peak area of each target compound to its internal standard (as the y-axis) against the ratio of the target compound concentration to the internal standard concentration (as the x-axis). A standard deviation-based method is used to calculate LOD and LOQ. The lowest concentration of the standard working solution was diluted 10-fold, and parallel measurements were performed 10 times. The detection limit (LOD) of the method was determined as three times the standard deviation (SD) of the results, and the quantification limit (LOQ) was defined as ten times the SD.

To evaluate precision and accuracy, six sets of parallel samples were taken within a single day, and continuous injections were performed to calculate the relative standard deviation (RSD) between parallel groups to confirm within-day repeatability. Additionally, five consecutive days of experimental injections were carried out to assess day-to-day repeatability. The concentrations of the three cooling agents in samples at three levels (low, medium, and high) were measured before and after spiking, and recovery rates were calculated to verify the accuracy of the method.

### 2.9 Applicability test

To evaluate the practical applicability of the proposed method, four flavors of electronic vapor products (Fresh Menthol, Ice Lemon Menthol, Ice Mint, and Cool Mint) from RELX (China) were analyzed. The objective was to assess the presence of cooling agents in popular market flavors and to determine the total aerosol content of the three cooling agents, as well as the distribution between the gas and particle phases, to verify the method’s practicality.

## 3 Results and discussion

### 3.1 Pretreatment conditions optimization

**FIGURE 2:**
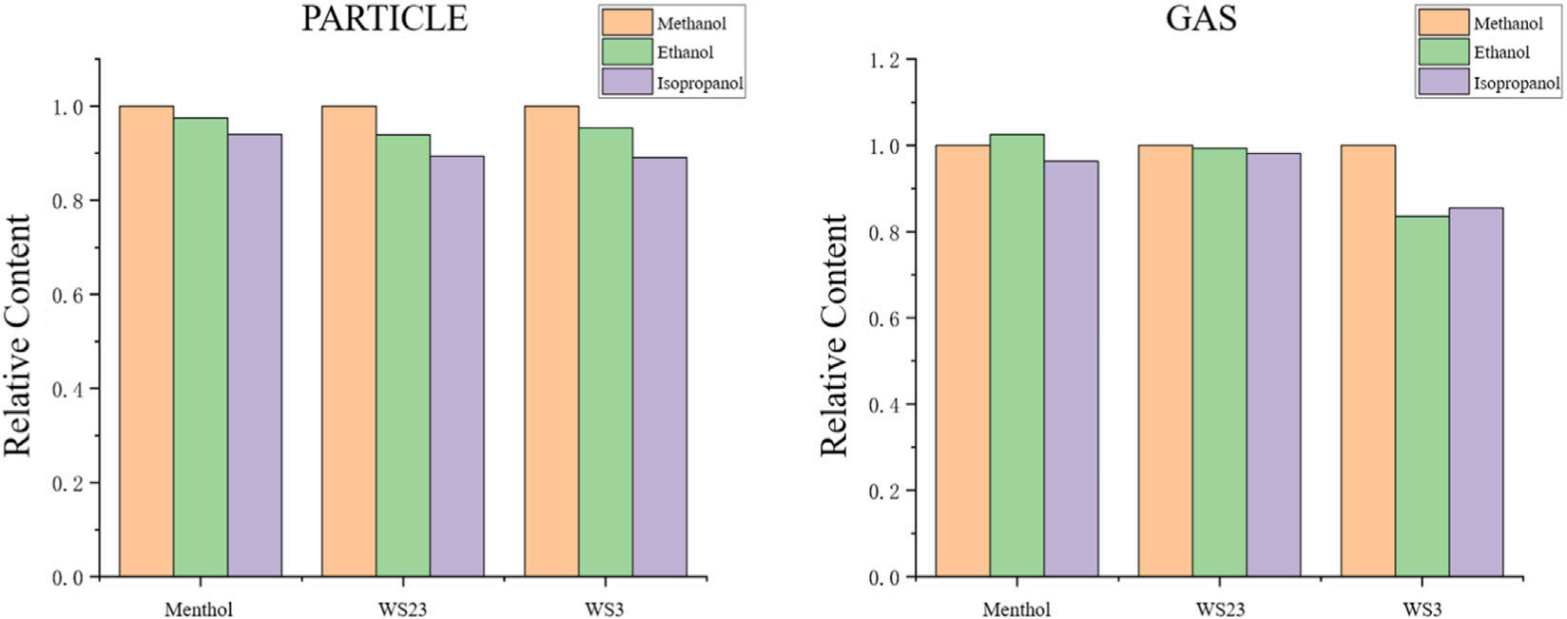
Relative contents of particle/gas-phase coolants under different solvents.

To balance the operation volume, time, and sample loss during the sample preparation process, the optimized processing conditions were compared and the best options were selected. This approach aimed to ensure complete extraction of the samples from the filter and adsorbent tubes, achieving the highest recovery rate. Based on the relative extraction efficiencies (Figure 2), methanol, which showed the best extraction performance, was chosen as the extraction solvent.

**FIGURE 3:**
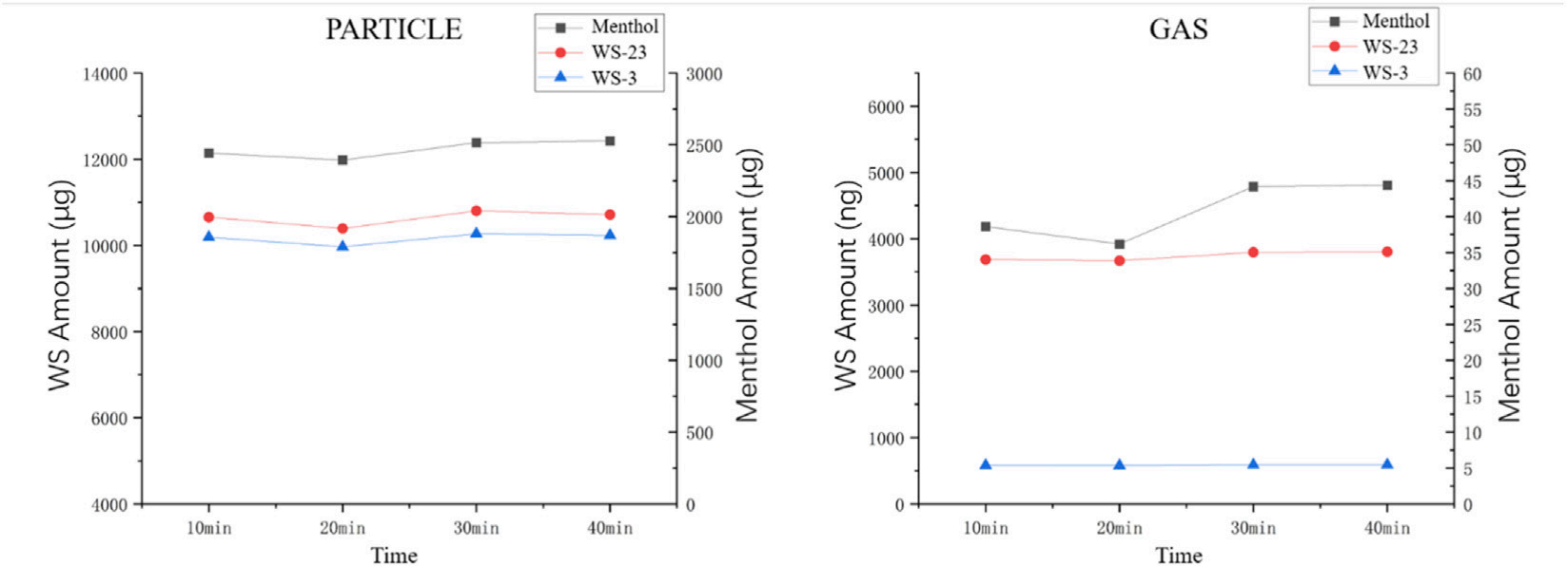
Particle/gas-phase coolants under different extraction times.

Insufficient extraction volumes may cause saturation, leading to incomplete sample extraction, while an excessive volume could increase the complexity of the procedure. After comparing three extraction volumes (Figure 3), 30 mL and 5 mL were selected for the particle phase and gas phase extraction volumes, respectively. At these volumes, the extraction yields reached near maximum levels, while also considering the convenience of handling gas phase samples (5 mL is easier to sample).

**FIGURE 4:**
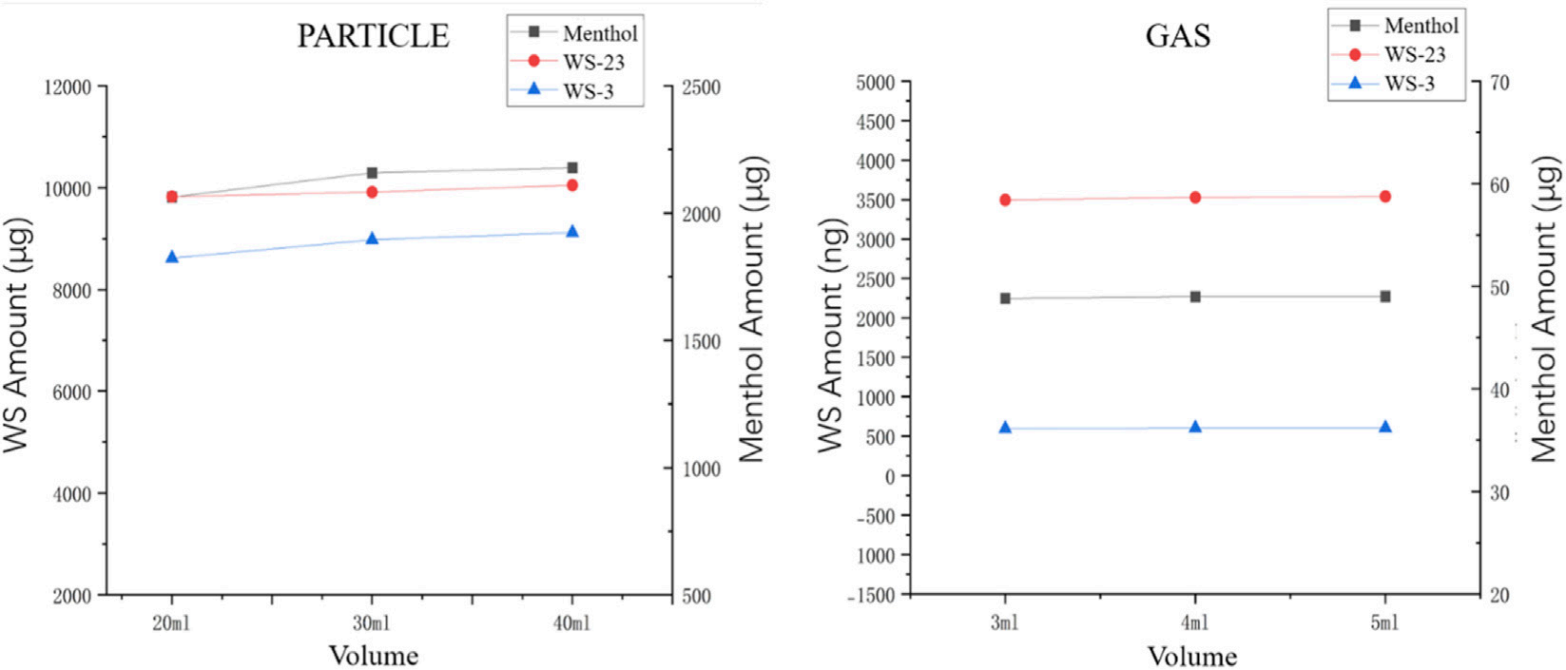
Particle/gas-phase coolants under different extraction solvent volumes.

During the optimization of ultrasonic conditions, different powers (40 W and 80 W) were tested, but no significant difference in extraction efficiency was observed. However, the extraction time did influence the extraction amount. As shown in Figure 4, the extraction reached its maximum after 30 min. It should be noted that during the extraction of gaseous menthol, the result after 20 min of extraction seemed to have decreased. Therefore, we calculated the deviation to be less than 5%, and considered that this slight fluctuation was caused by certain reasons. This fluctuation was within the acceptable range and did not affect the actual result. As a result, ultrasonic extraction conditions of 40W power and 30 min were chosen for extracting the aerosol cooling agents.

Thus, for the sample preparation of aerosol samples, 30 mL methanol was used for the particle phase and 5 mL methanol for the gas phase, with ultrasonic extraction at 40 W for 30 min, ensuring both high extraction efficiency and a reasonable operational timeframe.

### 3.2 Instrumental analysis methods optimization

To optimize the chromatographic peak shapes, the influence of different split ratios (splitless, 10:1, 20:1, 30:1) was evaluated. When the sample was analyzed without splitting, severe tailing and flat-top peaks were observed in the particle phase samples. As the split ratio increased, the tailing phenomenon was reduced; however, due to the lower concentration of the gas phase sample, signal intensity significantly decreased at higher split ratios. Considering both signal strength and peak shape quality, a split ratio of 10:1 was selected.

**FIGURE 5:**
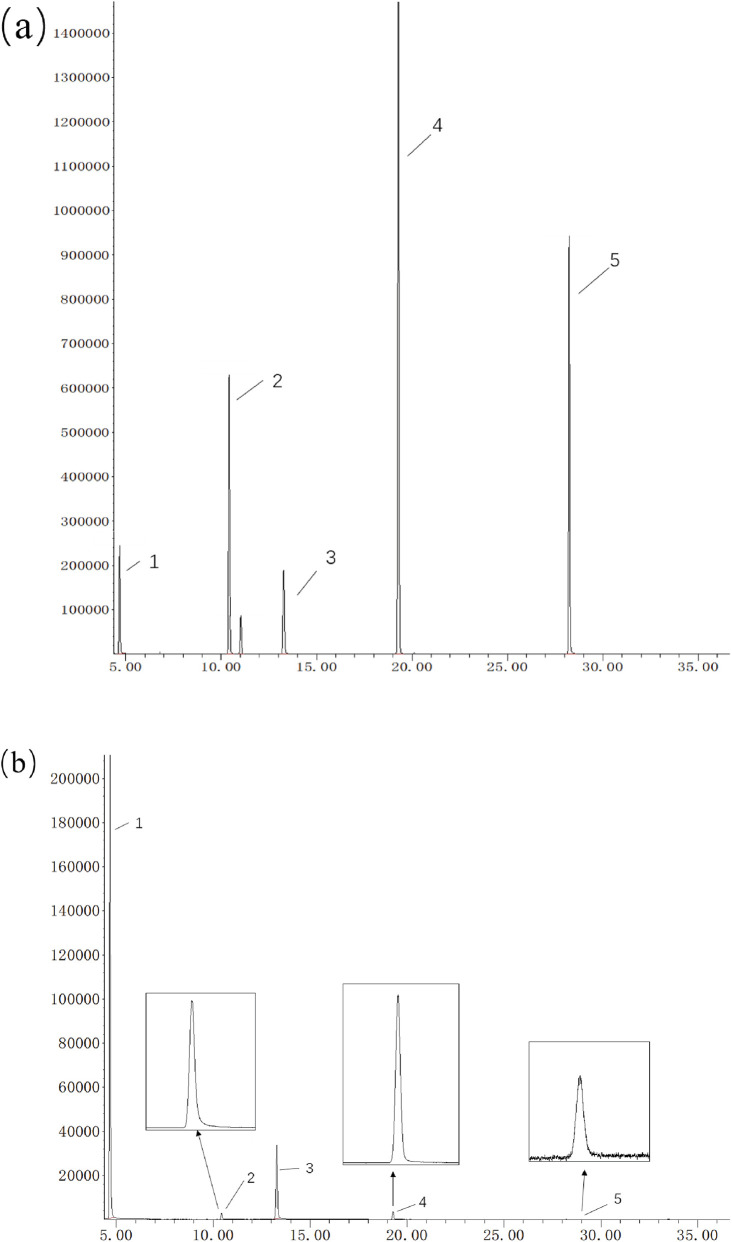
Chromatogram of particle/gas-phase aerosol samples under optimized GC–MS conditions; chromatogram of gas-phase aerosol samples under optimized GC–MS conditions. **(a)** particle, **(b)** gas.

Further optimization was performed on the temperature program’s heating rate and retention time. After balancing the separation of components and the required analysis time, the final temperature program was determined. Under the optimized chromatographic conditions, both internal standards and three cooling agents were well separated on the ALC-1 column, meeting the analytical requirements. The chromatogram of the smoke sample under optimized conditions is shown in Figure 5.

### 3.3 Adsorption tubes selection

Considering that different types of adsorbent tubes may have varying adsorption capacities for gas-phase cooling agents, this study selected three models of adsorbent tubes: ORBO™615, ORBO™32, and ORBO™90, for comparative evaluation. Due to the low concentration of target compounds in the aerosolized samples, it is challenging to effectively assess the adsorption and desorption efficiency of the adsorbent tubes. Therefore, thermogravimetric analysis was used to select the optimal adsorbent tube and evaluate its performance for capturing gas-phase cooling agents.

**FIGURE 6:**
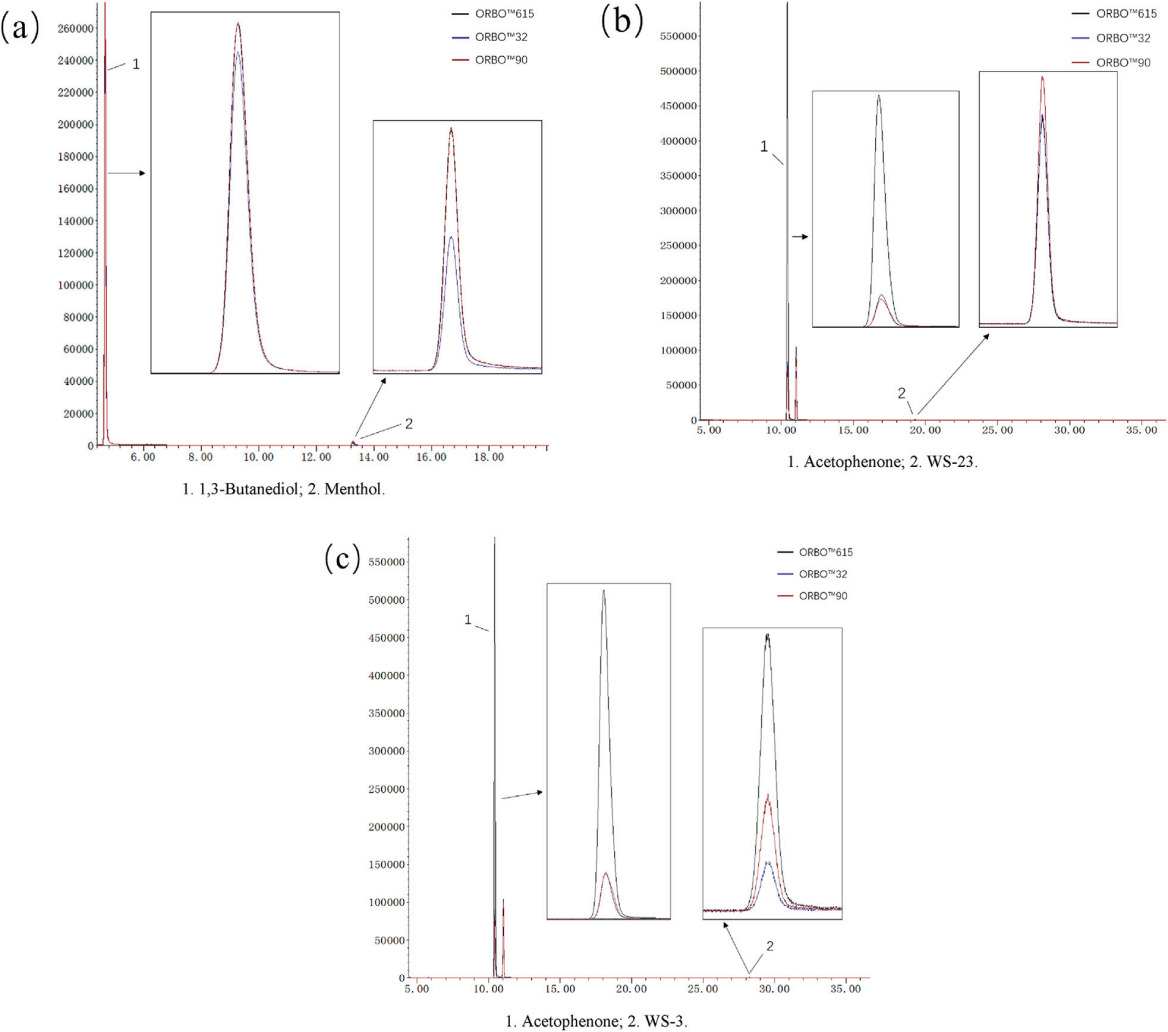
Comparative chromatograms of adsorbent tubes. **(a)** menthol; **(b)** ws-23; **(c)** ws-3.

The results, shown in Figure 6, led to the following conclusions: (1) The response for menthol in the ORBO™32 tube was significantly lower than in the other two models (Figure 6a). This is likely due to the weaker adsorption and desorption capacity of the coconut shell activated carbon filling in the ORBO™32 tube for both the 1,3-butanediol internal standard and menthol. (2) For WS-23 and WS-3, both of which are amide compounds, phenylethanone-d8 was used as the internal standard. The chromatographic responses (Figures 6b,c) showed that the XAD-7 resin filling in the ORBO™615 tube exhibited excellent adsorption capacity for these compounds, with high desorption efficiency in methanol solution. The chromatographic response signals were clearly superior to those from the other two adsorbent tubes. In contrast, the signals for phenylethanone-d8 and the target compounds in the ORBO™90 and ORBO™32 tubes showed significant attenuation, indicating poor adsorption and desorption efficiency for both the cooling agents and the internal standard.

In summary, based on the adsorption and desorption efficiency for the three cooling agents, the ORBO™615 adsorbent tube was selected as the optimal device for capturing gas-phase components in electronic cigarette aerosols.

To further verify the reliability of the ORBO™615 adsorbent tube in capturing gas-phase cooling agents, a tandem adsorption experiment was conducted. Two ORBO™615 tubes (Adsorbent 1&2 in Table 3) were connected in series to collect aerosol samples, and the target compound contents in the front and rear tubes were measured separately to determine whether any breakthrough occurred. In addition, to evaluate the completeness of ultrasonic extraction, samples were analyzed after 30 min of ultrasonic extraction, and then again after extending the extraction time to 40 and 50 min. The results were compared to theoretical spiked amounts to calculate recovery rates, which ranged from 89.4% to 98.56%.

**TABLE 3:** Gas-phase cooling agent distribution ratios and recovery efficiencies in adsorbent tubes.

Compounds	Adsorption Quantity (μg)		Amount (μg)	Recovery
	Adsorbent 1	Adsorbent 2		
Menthol	20.698	N.D.	21	98.56%
WS-23	16.685	N.D.	18	92.69%
WS-3	11.594	N.D.	13	89.48%

N.D., not detected.

**FIGURE 7:**
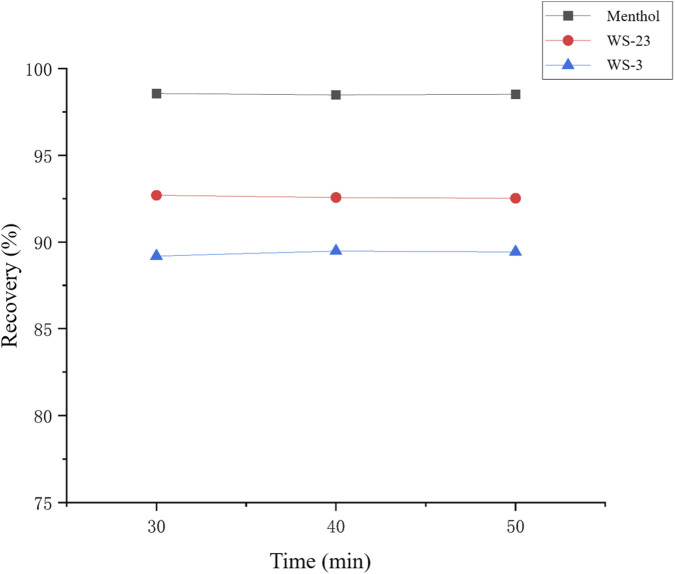
Verification of extraction time for ORBO™615 adsorbent tubes.

**FIGURE 8:**
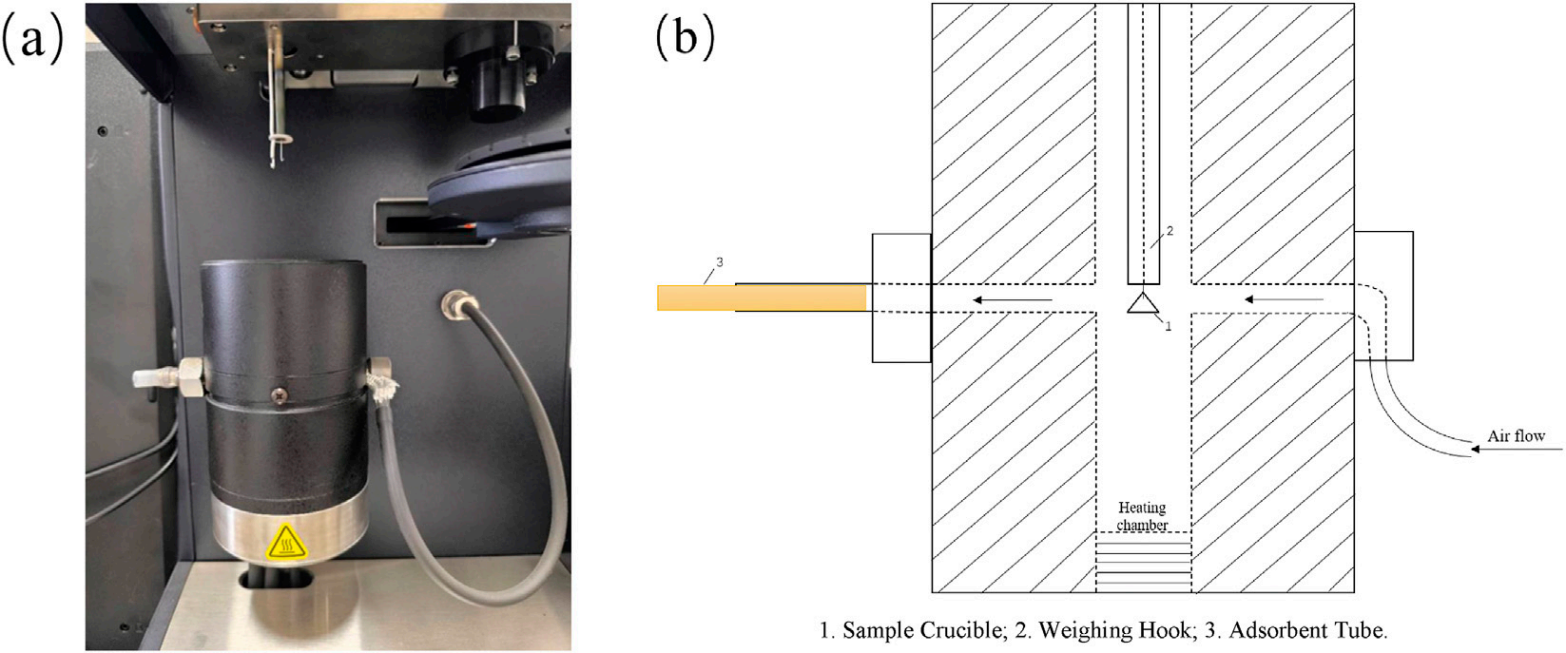
Thermal Gravimetric analyzer. **(a)** Chamber section, **(b)** schematic diagram of chamber section.

The results showed that, in the tandem adsorption experiment, the ORBO™615 tube demonstrated high adsorption efficiency for all three cooling agents, with no breakthrough observed. The recoveries of the three cooling agents ranged from 89.48% to 98.56% (Table 3), and extending the extraction time did not significantly improve recovery (Figure 7). Notably, WS-23 and WS-3 exhibited slightly lower recoveries (approximately 90%). This is likely due to their low vapor pressures (Table 4) and the dead volume in the thermogravimetric analyzer chamber (Figure 8). Specifically, during heating, the vaporized analytes were transported by the purge gas to the adsorbent tube; however, upon leaving the vertical heating chamber, the gas temperature dropped rapidly, causing a small fraction of gas-phase cooling agents to condense into the solid phase and remain in the connecting tubing.

**TABLE 4:** Vapor pressures and boiling points of cooling agents.

Compounds	Vapor pressure (Pa)	Boiling point (°C)
Menthol	106.6 (20 °C)	216
WS-23	0.3 (25 °C)	255
WS-3	0.007 (25 °C)	351

In conclusion, the ORBO™615 adsorbent tube demonstrates excellent adsorption capacity and recovery performance for gas-phase menthol, WS-3, and WS-23 in electronic cigarette aerosols, making it suitable for subsequent analytical applications.

### 3.4 Method validation

#### 3.4.1 Calibration curves, limits of Detection (LOD), and limits of Quantitation (LOQ)

The linear ranges, regression equations, correlation coefficients, limits of detection (LOD), and limits of quantification (LOQ) are summarized in Tables 5, 6. All calibration curves exhibited correlation coefficients (*R* ^2^) ≥ 0.9994, indicating excellent linearity. The LODs and LOQs of the method were 0.051–0.255 μg/mL and 0.169–0.849 μg/mL, respectively.

**TABLE 5:** Linear range, regression equation, correlation coefficient, LOD, and LOQ of particle-phase targets.

Compounds	Range (μg/mL)	Calibration equations	*R* ^2^	LOD (μg/mL)	LOQ (μg/mL)
Menthol	10–500	Y = 1.447752X+0.031370	0.9996	0.051	0.169
WS23	10–500	Y = 0.750296X+0.029708	0.9994	0.114	0.380
WS3	10–500	Y = 0.740571X-0.073878	0.9998	0.082	0.273

**TABLE 6:** Linear range, regression equation, correlation coefficient, LOD, and LOQ of gas-phase targets.

Compounds	Range (ng/mL)	Calibration equations	*R* ^2^	LOD (ng/mL)	LOQ (ng/mL)
Menthol	10–1,000	Y = 5.586838X+0.038901	0.9997	0.255	0.849
WS23	10–1,000	Y = 0.447089X-0.002682	0.9999	0.137	0.456
WS3	10–1,000	Y = 0.180606X-0.0007033	0.9997	0.175	0.585

To evaluate the precision and accuracy of the established method, intra-day precision, inter-day precision, and spiked recovery experiments were conducted. For intra-day precision, six parallel samples were analyzed consecutively, and the relative standard deviations (RSDs) of the target compounds among the replicates were calculated. For inter-day precision, the same experiment was repeated over five consecutive days. Spiked recovery was determined by adding a mixed standard solution of cooling agents at low, medium, and high concentration levels to gas-phase and particle-phase samples of known content, followed by measurement of the cooling agent concentrations before and after spiking.

As shown in Tables 7, 8, the intra-day RSDs for the three cooling agents were ≤3.32%, and the inter-day RSDs were ≤4.15%, demonstrating good repeatability and stability of the method. The recoveries at low, medium, and high levels were 92.93%–113.25%, 94.08%–109.78%, and 91.32%–109.36%, respectively, all within the acceptable range. It should be noted that we calculated recoveries using a calibration curve prepared in pure solvent (neat standard). Thus, the measured recoveries can be taken as approximate matrix factor (MF) values between ∼91% and ∼113%, which is modest but is not a formal matrix effect study. Overall, the developed method exhibited excellent precision and reproducibility, and is suitable for accurate quantification of both gas-phase and particle-phase menthol, WS-3, and WS-23 in electronic cigarette aerosols.

**TABLE 7:** Precision and recovery of particle-phase targets in aerosols.

Compounds	Intra-day (RSD%)	Inter-day (RSD%)	Accuracy (%)		
			Low level	Medium level	High level
Menthol	1.40%	3.65%	106.26%	109.15%	107.89%
WS23	2.87%	2.27%	113.25%	109.53%	99.19%
WS3	2.94%	3.90%	112.95%	109.78%	99.83%

**TABLE 8:** Precision and recovery of gas-phase targets in aerosols.

Compounds	Intra-day (RSD%)	Inter-day (RSD%)	Accuracy (%)		
			Low level	Medium level	High level
Menthol	1.54%	4.15%	105.29%	107.27%	109.36%
WS23	3.21%	3.16%	104.80%	106.14%	108.61%
WS3	3.32%	4.06%	92.93%	94.08%	91.32%

#### 3.4.2 Applicability test

**FIGURE 9:**
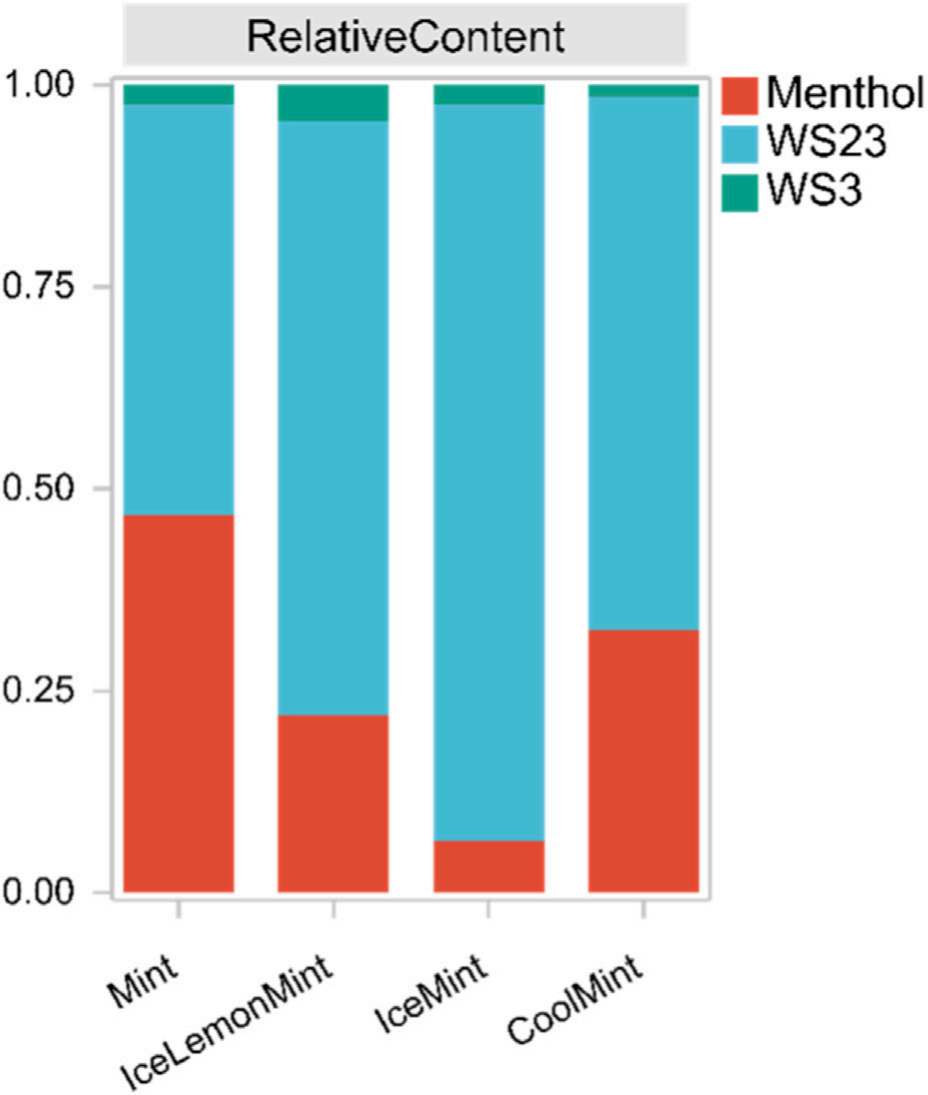
Relative contents of cooling agents in aerosols from different samples.

To verify the practical applicability of the developed method, four commercially available electronic cigarette products with different flavors were analyzed to determine the concentrations of three cooling agents in their aerosols, as shown in Tables 9, 10. Quantitative results indicated that the total contents of the cooling agents varied considerably among products due to differences in e-liquid formulations. However, the gas/particle-phase distribution ratios for each cooling agent showed no significant variation, suggesting that the distribution was primarily governed by the inherent volatility of the compounds. As illustrated in Figure 9, all three cooling agents were detected in the aerosols, and their proportions were largely determined by the levels added to the e-liquid. In all four products, WS-23 was present at the highest concentration, whereas WS-3 was the lowest. This pattern is consistent with previous sensory studies (Behrendt et al., 2004; Johnson et al., 2018), which reported a cooling intensity ranking of WS-3 > menthol > WS-23. Consequently, the amount added in the formulations appears to be inversely correlated with the intrinsic cooling strength of each agent.

**TABLE 9:** Contents of three cooling agents in aerosols of different E-cigarette samples.

Samples	Menthol		WS-23		WS-3	
	Particle (μg·puff^−1^)	Gas (μg·puff^−1^)	Particle (μg·puff^−1^)	Gas (μg·puff^−1^)	Particle (μg·puff^−1^)	Gas (μg·puff^−1^)
Menthol	141.812	7.284	162.411	0.082	7.655	0.014
Ice Lemon Menthol	71.222	4.318	253.286	0.196	15.224	0.024
Ice Mint	37.381	0.741	548.413	0.212	14.397	0.014
Cool Mint	176.822	4.472	367.581	0.106	7.685	0.012

**TABLE 10:** Gas–particle partition ratios of three cooling agents in aerosols of different E-cigarette samples.

Samples	Menthol		WS-23		WS-3	
	Particle	Gas	Particle	Gas	Particle	Gas
Menthol	95.11%	4.89%	99.95%	0.05%	99.82%	0.18%
Ice Lemon Menthol	94.28%	5.72%	99.92%	0.08%	99.84%	0.16%
Ice Mint	98.06%	1.94%	99.96%	0.04%	99.90%	0.10%
Cool Mint	97.53%	2.47%	99.97%	0.03%	99.85%	0.15%

The phase distribution of cooling agents in aerosols reflects a dynamic equilibrium between gas-phase and particle-phase, which is directly related to compound volatility (Pankow, 2001). The results showed that all three cooling agents predominantly existed in the particle phase, with the gas-phase fraction being relatively small and following the trend: menthol > WS-3 > WS-23. Menthol, being the most volatile, exhibited the highest gas-phase proportion. Although WS-23 and WS-3 are both amide-type cooling agents and WS-23 has a higher vapor pressure and lower boiling point, its gas-phase proportion was slightly lower than that of WS-3. This phenomenon may be attributed to the higher WS-23 concentration in e-liquids, leading to a greater fraction condensing into the particle phase during aerosol cooling, thereby reducing its relative gas-phase proportion. To verify this hypothesis, an additional e-liquid was prepared with equal concentrations of WS-23 and WS-3. When the generated aerosol masses of the two were comparable, the gas-phase fraction of WS-23 exceeded that of WS-3, consistent with their boiling point and vapor pressure characteristics.

In summary, analysis of the four market-available flavors demonstrated that the developed method is effective for determining cooling agents in electronic cigarette aerosols. Moreover, the observed phase distributions appear to follow predictable physicochemical property trends, providing a basis for future investigations into the sensory effects, sensory thresholds, and exposure risks associated with different phase distributions.

## 4 Conclusion

In this study, a high-efficiency analytical method was developed to determine three representative cooling agents—menthol, WS-23, and WS-3—in aerosols by GC–MS via systematic optimization of sample pretreatment parameters including extraction solvent type, extraction time, and solvent volume. The method features a simple pretreatment procedure, high detection sensitivity, and a short analysis time. Experimental results indicated that the concentrations of cooling agents in the aerosol gas phase were substantially lower than those in the particle phase, with gas-phase proportions of 1.94%–5.72% for menthol, 0.03%–0.08% for WS-23, and 0.10%–0.18% for WS-3. All three cooling agents predominantly existed in the particle phase, and their gas-phase distribution was jointly influenced by physicochemical properties such as boiling point and vapor pressure. The phase differentiation values obtained by this method have important implications for improving the accuracy of inhalation exposure assessment, as previous biomimetic and numerical simulation studies have demonstrated that aerosol particle size is directly associated with deposition sites and deposition efficiency in the respiratory tract (Ciloglu and Karaman, 2022; Koullapis et al., 2016; Kumar and Singh, 2023; Li et al., 2021). Consequently, this method provides a reliable analytical tool for investigating gas–particle partitioning behavior of typical cooling agents in electronic cigarettes, and provides experimental evidence and data to support further studies on their deposition characteristics in the human respiratory system and the resulting sensory effects.

## Data Availability

The original contributions presented in the study are included in the article/supplementary material, further inquiries can be directed to the corresponding authors.
